# LipidCruncher: an open-source platform for processing, visualizing, and analyzing lipidomic data

**DOI:** 10.1186/s12859-026-06483-3

**Published:** 2026-06-04

**Authors:** Hamed Abdi, Yohannes A. Ambaw, Zon Weng Lai, Ritchie Ly, Chandramohan Chitraju, Shubham Singh, Robert V. Farese, Tobias C. Walther

**Affiliations:** 1 Cell Biology Program, Sloan Kettering Institute, New York, NY USA; 2 Present Address: mRNA Center of Excellence, Sanofi, Waltham, MA USA; 3 Howard Hughes Medical Institute, New York, NY USA

**Keywords:** Lipids, Lipidomics, Sterols, Sphingolipids, Phospholipids, Bioinformatics, Computational biology, Mass spectrometry, Open-source software, Scientific software

## Abstract

**Background:**

Advances in mass spectrometry (MS)-based lipidomics have led to a significant surge in data volume, underscoring a need for robust tools to efficiently evaluate and visualize these expansive datasets. While numerous software tools have been developed, current workflows are hindered by manual spreadsheet handling and insufficient data quality assessment prior to analysis. Here, we introduce *LipidCruncher*, an open-source, web-based platform designed to easily process, visualize, and analyze lipidomic data with high efficiency and rigor.

**Results:**

*LipidCruncher* consolidates key steps of the lipidomics analysis workflow, including data standardization, normalization, and stringent quality controls. The platform also provides advanced visualization and analysis tools that are tailored to interrogate lipidomic data and enable detailed and holistic data exploration. To illustrate *LipidCruncher*’s utility, we analyzed lipidomic data from adipose tissue of mice lacking the triacylglycerol synthesis enzymes DGAT1 and DGAT2.

**Conclusions:**

*LipidCruncher* fills a specific gap in the lipidomics analysis ecosystem by providing an integrated, quality-focused platform that accepts data from multiple sources and complements existing specialized tools. By bridging the critical divide between data generation and biological interpretation, *LipidCruncher* facilitates rigorous lipidomics analyses to accelerate the translation of complex lipid profiles into biological insights.

**Supplementary Information:**

The online version contains supplementary material available at 10.1186/s12859-026-06483-3.

## Background

Lipids include diverse classes and thousands of lipid species [1, 2], presenting challenges in identifying changes under different physiological conditions. Despite these complexities, quantification and characterization of lipids are essential for dissecting metabolic and signaling pathways involving lipids.

Mass spectrometry (MS) applied to lipid analyses, either via liquid chromatography coupled to mass spectrometry (LC-MS) or “shotgun” approaches, enable collection of large datasets with quantitation of hundreds to thousands of lipid species [3–5]. Analysis of the resultant lipidomic datasets is challenging and involves several steps. First, mass spectra are processed using specialized software (e.g., *LipidSearch* [6], *LipidXplorer* [7], *LipidFinder* [8] and *MS-DIAL* [9]) that assigns specific lipid species to ion current peaks at particular mass-to charge ratios and quantifies their abundance, while utilizing fragmentation spectra of corresponding ions to enhance identification accuracy and structural characterization. These tools turn mass spectra into a structured dataset that can be the starting point for subsequent bioinformatic analyses.

Other software tools (e.g., *lipidr* [10], *LipidSig* [11], *ADViSELipidomics* [12], *MetaboAnalyst* [13], *LipidOne* [14], *MZmine* [15], and *Lipostar* [16]) provide sophisticated features for processing, visualizing, and analyzing lipidomic data, with several offering capabilities that span from raw data processing to biological interpretation. While each of these tools has particular strengths, an accessible platform that accepts native data input from major lipidomics pipelines, integrates quality control tightly with normalization, and offers comprehensive lipid-centric visualization in a guided workflow would be of great utility.

To address these challenges, we developed *LipidCruncher*, an open-source, user-friendly web-based tool that accommodates diverse data formats and streamlines the transition from semi-processed data to lipid-centric data analysis and visualizations. *LipidCruncher* simplifies and standardizes the workflow, integrating a robust framework for data standardization and normalization with rigorous quality checks to ensure the integrity and reliability of the analysis. Rather than replicating the breadth of general-purpose analytics platforms, *LipidCruncher* provides a guided, quality-focused workflow specifically optimized for biologists working with processed lipidomic data. Its suite of interactive visualizations provides an intuitive interface that complements existing specialized tools and facilitates biological interpretation.

## Results

### Description of LipidCruncher workflow

**Fig. 1:**
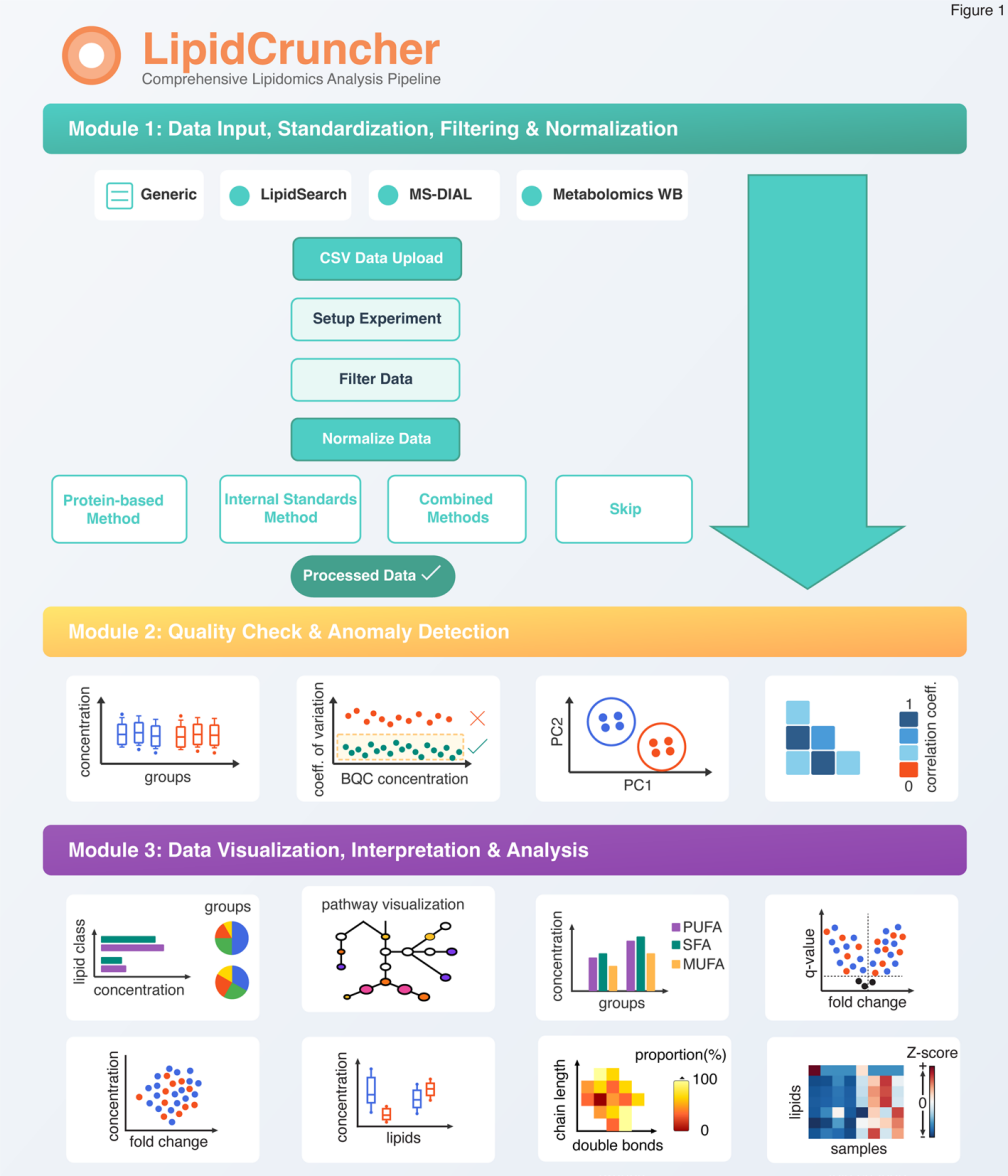
Overview of the *LipidCruncher* analysis pipeline. The workflow is organized into three modules. Module 1 handles data standardization, filtering, and normalization. Module 2 assesses data quality: box plots verify concentration distribution uniformity across replicates, coefficient of variation (CoV) analysis of batch quality control (BQC) samples evaluates measurement precision, and correlation heatmaps and principal component analysis (PCA) enable outlier detection through replicate comparison and sample clustering visualization. Module 3 provides visualization tools: bar and pie charts display lipid class concentration and proportions, metabolic network visualization contextualizes changes across lipid classes, saturation profiles show fatty acid composition (SFA, MUFA, PUFA), volcano plots highlight statistically significant changes with accompanying mean concentration versus fold-change plots and box plots for selected lipids, fatty acid composition heatmaps reveal shifts in chain length and saturation, and comprehensive lipidomic heatmaps with hierarchical clustering display patterns across all detected species

The workflow and features of *LipidCruncher* are organized into three modules (Fig. 1). The following sections summarize the key capabilities of each module; detailed technical specifications and procedures are provided in Supplementary Methods.

#### Module 1: data input, standardization, filtering, and normalization

The first step is data upload (Module 1 of Fig. 1), for which *LipidCruncher* accommodates outputs from various software platforms. Users can provide data in a generic format (Supplementary File 1) or import datasets from *LipidSearch* (Supplementary File 2) [6], *MS-DIAL* (Supplementary File 3) [9], or *Metabolomics Workbench* (Supplementary File 4) [17]. These formats cover widely used platforms in the lipidomics community, including both commercial (*LipidSearch*) and open-source (*MS-DIAL*) mass spectrometry processing software, while also enabling access to publicly available datasets from *Metabolomics Workbench* and providing a generic option for compatibility with any processing pipeline. For the generic format, input data consist of a column for lipid species names accompanied by columns containing abundance values for each sample (Supplementary File 1). *Metabolomics Workbench* files follow a similar structure to the generic format but include additional metadata such as sample information and experimental conditions. Input files from *LipidSearch* [6] or *MS-DIAL* [9] contain additional data columns (calculated mass, retention time, quality grades) that enable quality assessment features that are not possible with the generic format (Supplementary Methods S1.1–S1.4).

Upon data upload, *LipidCruncher* standardizes column naming, removes duplicates and invalid entries and replaces null or negative values with zeros (Supplementary Methods S2.1–S2.2). Zero-filtering removes lipid species with excessive zero or below-detection intensity values based on user-configurable thresholds. A species is retained only if it passes the BQC threshold (when BQC samples are present) and at least one non-BQC condition (Supplementary Methods S2.4). *LipidCruncher* does not impute missing values, as zero values in lipidomics frequently represent true biological absence rather than random technical missingness, and standard imputation methods can introduce systematic bias in such cases [18]. For analyses requiring log transformation, zeros are replaced with one-tenth of the smallest non-zero value in the dataset to maintain computational integrity (Supplementary Methods S2.5).

For *LipidSearch* [6] and *MS-DIAL* [9] inputs, quality-based filtering is also applied. *LipidCruncher* provides five normalization options: (1) no normalization for pre-normalized datasets, (2) internal standard-based normalization, (3) protein-based normalization, or (4) combined normalization using both standards and protein-based methods, or (5) total intensity normalization [19, 20]. For internal standard based normalization, *LipidCruncher* automatically detects common standards including SPLASH LIPIDOMIX^®^ and deuterated labels, or users can upload custom standard lists. Before normalization, the platform generates diagnostics bar plots displaying raw intensity values of each internal standard across all samples. Uniform bar heights indicate consistent sample preparation and instrument performance (Supplementary Methods S3.1–S3.4).

#### Module 2: quality check and anomaly detection

Assessing the quality of the data is a crucial prerequisite step to processing lipidomic data. After normalization, *LipidCruncher* provides several diagnostic tools to assess data quality (Module 2 of Fig. 1). Box plots display concentration distributions for each sample, where replicates from the same condition should exhibit similar medians and interquartile ranges. A bar chart quantifies the percentage of zero values per sample to identify anomalies. For datasets with batch quality control (BQC) samples—technical replicates created by pooling equal aliquots from all experimental samples—the coefficient of variation (CoV) is calculated for each lipid species. Users can optionally filter species exceeding a chosen threshold (default 30%, Supplementary Methods S4.1–S4.2). Removed species are displayed, allowing users to selectively restore lipids of biological interest before finalizing.

The dataset is further evaluated using pairwise correlation and principal component (PCA) analyses [21]. Correlation heatmaps reveal deviations from expected patterns that may indicate sample outliers (Supplementary Methods S4.3). PCA reduces dataset dimensionality and visualizes replicate clustering, with 95% confidence ellipses drawn around each experimental condition (Supplementary Methods S4.4). Samples falling outside these ellipses are flagged as potential outliers. When anomalies are identified, *LipidCruncher* provides the option to exclude aberrant samples.

#### Module 3: data visualization, interpretation, and analysis

This module provides interactive visualizations for exploring lipidomic data (Module 3 of Fig. 1**)**. All plots allow users to hover over data points to reveal more detailed information.

*LipidCruncher* generates bar plots displaying mean lipid class concentrations across conditions with standard deviation error bars and statistical significance indicators. Complementary pie charts illustrate proportional distributions of lipid classes. To facilitate detection of altered metabolic pathways, *LipidCruncher* visualizes lipid abundances in a metabolic network context. The platform provides options for 28 curated lipid classes connected by biologically relevant metabolic edges, with options to start from a default 18-class pathway, all 28 classes, or an empty canvas. Circle size reflects the concentration fold-change between conditions and color indicates the fatty acid saturation ratio. The layout is fully customizable, and configurations can be saved and reloaded across sessions (Supplementary Methods S6.5). Separately, fatty acid saturation profiles present the composition of saturated (SFA), mono-unsaturated (MUFA), and poly-unsaturated (PUFA) fatty acids within each lipid class through two complementary views: bar plots of absolute concentrations and stacked bar charts of relative percentages (Supplementary Methods S6.4).

Volcano plots display log2 fold-change against statistical significance, highlighting significant changes in the upper and outer quadrants. Users can customize thresholds, filter lipid classes, choose statistical tests, and apply multiple testing corrections. Complementary plots show fold-change versus mean concentration and box plots for selected lipids.

*LipidCruncher* offers two types of heatmaps. Fatty acid composition heatmaps [22] display lipids within a selected class by double bond count (x-axis) and carbon chain length (y-axis). Color intensity indicates each species’ relative abundance within the class (Supplementary Methods S6.3). Multiple conditions are shown side-by-side with average double bond and chain length markers overlaid, enabling detection of compositional shifts. Comprehensive lipidomic heatmaps display all lipids (rows) across samples (columns) with z-score color coding. Users can preserve original lipid order or apply Ward’s hierarchical clustering [23] to group lipids with similar concentration patterns (Supplementary Methods S6.1**–**S6.2).

For statistical comparisons, the platform uses Welch’s t-test for two conditions or Welch’s ANOVA with post-hoc testing for multiple conditions, with options for non-parametric alternatives (Mann-Whitney U, Kruskal-Wallis) [24–27].* P*-values are adjusted using Benjamini-Hochberg or Bonferroni methods [28], with a two-level framework controlling false discoveries both across lipid classes and within pairwise comparisons (Supplementary Methods S5.1–S5.2).

For data export, users can download plot data in CSV format or visualizations as SVG files. *LipidCruncher* generates comprehensive PDF reports including all visualizations. While designed for lipidomics, the core processing, quality control, and general visualization features (e.g., bar charts, box plots, PCA, correlation analysis, hierarchical clustering, volcano plots) are applicable to other quantitative omics datasets formatted into the generic CSV input.

### Case study example of utilizing LipidCruncher to analyze a lipidomic dataset

#### Data input and data quality analysis for the case study

**Fig. 2:**
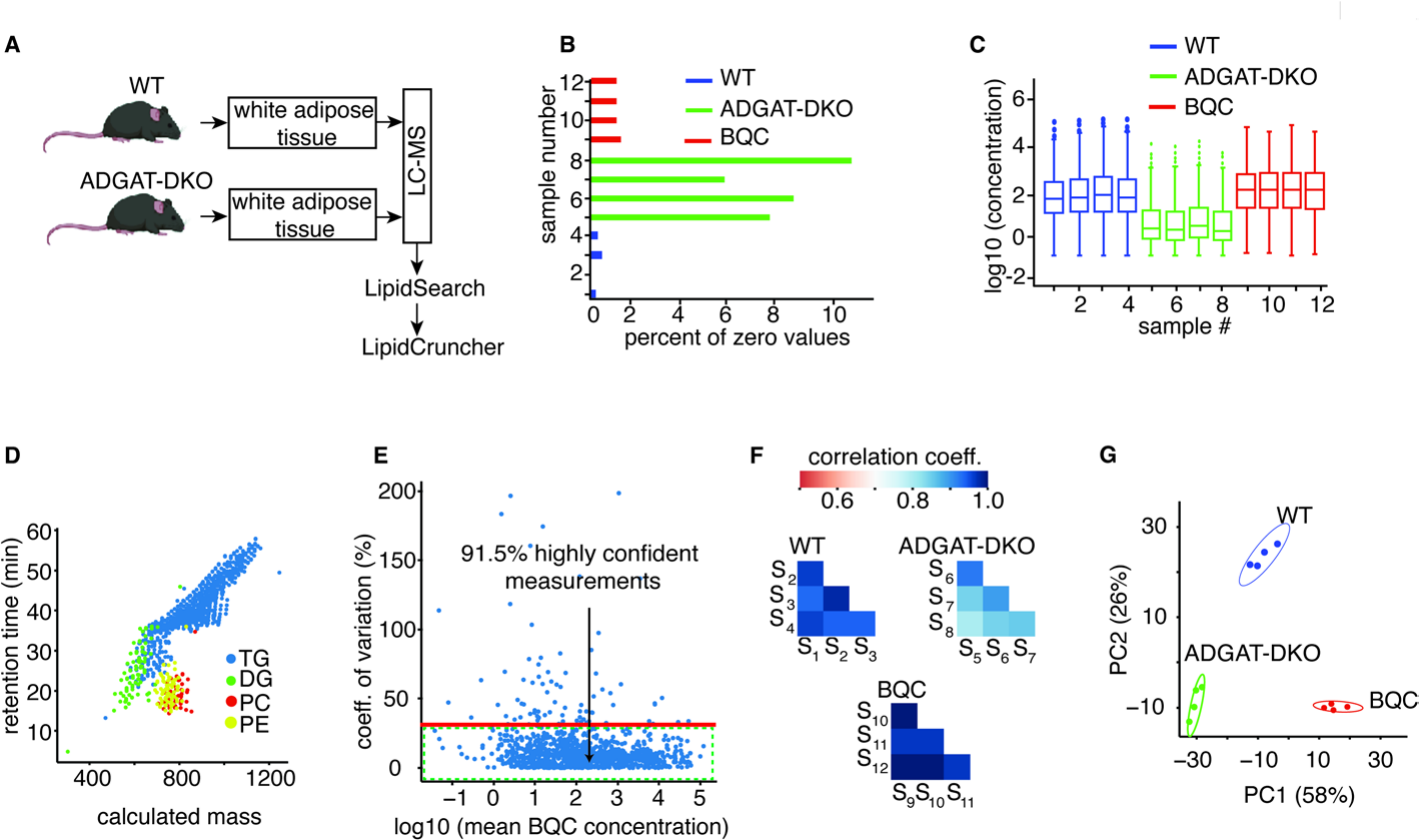
Quality check analysis of lipidomic dataset from ADGAT-DKO mice. **A** Experimental design. **B** Bar plot showing the percentage of zero values per sample. **C** Box plots displaying normalized lipid concentration distributions across samples. **D** Scatter plot of retention time versus calculated mass for representative lipid classes (TG, DG, PC, and PE), demonstrating expected clustering patterns based on hydrophobicity. **E** Scatter plot of Coefficient of Variation (CoV) against mean concentration for BQC samples. **F** Correlation heatmaps showing coefficients between replicates within WT and ADGAT-DKO conditions. **G** PCA plot showing clustering of WT and ADGAT-DKO samples with 95% confidence ellipses

To demonstrate the workflow and utility of *LipidCruncher*, we analyzed lipidomic data from murine adipose tissue in which the enzymes catalyzing triglyceride synthesis, DGAT1 and DGAT2, were deleted (ADGAT-DKO mice) [29] (Fig. 2A). The following analysis focuses on illustrating the platform’s capabilities; detailed biological interpretation of this dataset has been reported previously [29]. The study included WT and ADGAT-DKO groups, each with four biological replicates, plus four BQC samples. Lipid analysis was performed using LC-MS/MS following lipid extraction with the Folch method [30], with raw data processed using *LipidSearch* 5.0 [6], and the resultant CSV output files used as input for *LipidCruncher* (Supplementary File 2).

After data standardization and filtering, *LipidCruncher* generated a refined dataset containing 942 endogenous lipid species, spanning 15 lipid classes, along with nine internal standards used for normalization (Supplementary Methods S8.1–S8.3).

Analysis of the dataset revealed a higher proportion of zero values in the ADGAT-DKO samples (~ 8%) than WT samples (~ 0.4%) (Fig. 2B), indicating substantial lipidomic changes due to DGAT deletion. Box plots confirmed uniform concentration distributions across replicates within each condition (Fig. 2C**)**, validating the normalization process. The retention time versus calculated mass plot demonstrated expected clustering patterns for lipid classes based on their hydrophobicity, with TGs displaying the longest retention times and phospholipids (PC, PE) showing shorter retention times **(**Fig. 2D**)**. This plot serves as a quality check for lipid identification consistency; deviations from expected cluster positions would indicate potential misidentifications.

CoV analysis showed that > 90.0% of lipid species had CoV below 30% across BQC samples (Fig. 2E); species exceeding this threshold were removed. Strong correlations (> 0.8) between replicates confirmed data consistency (Fig. 2F). PCA plots showed distinct clustering within 95% confidence ellipses for each condition (Fig. 2G), with clear separation between WT and ADGAT-DKO groups reflecting the substantial lipidomic differences. No anomalies were detected during quality assessment.

#### Data visualization, interpretation, and analysis for the case study

**Fig. 3:**
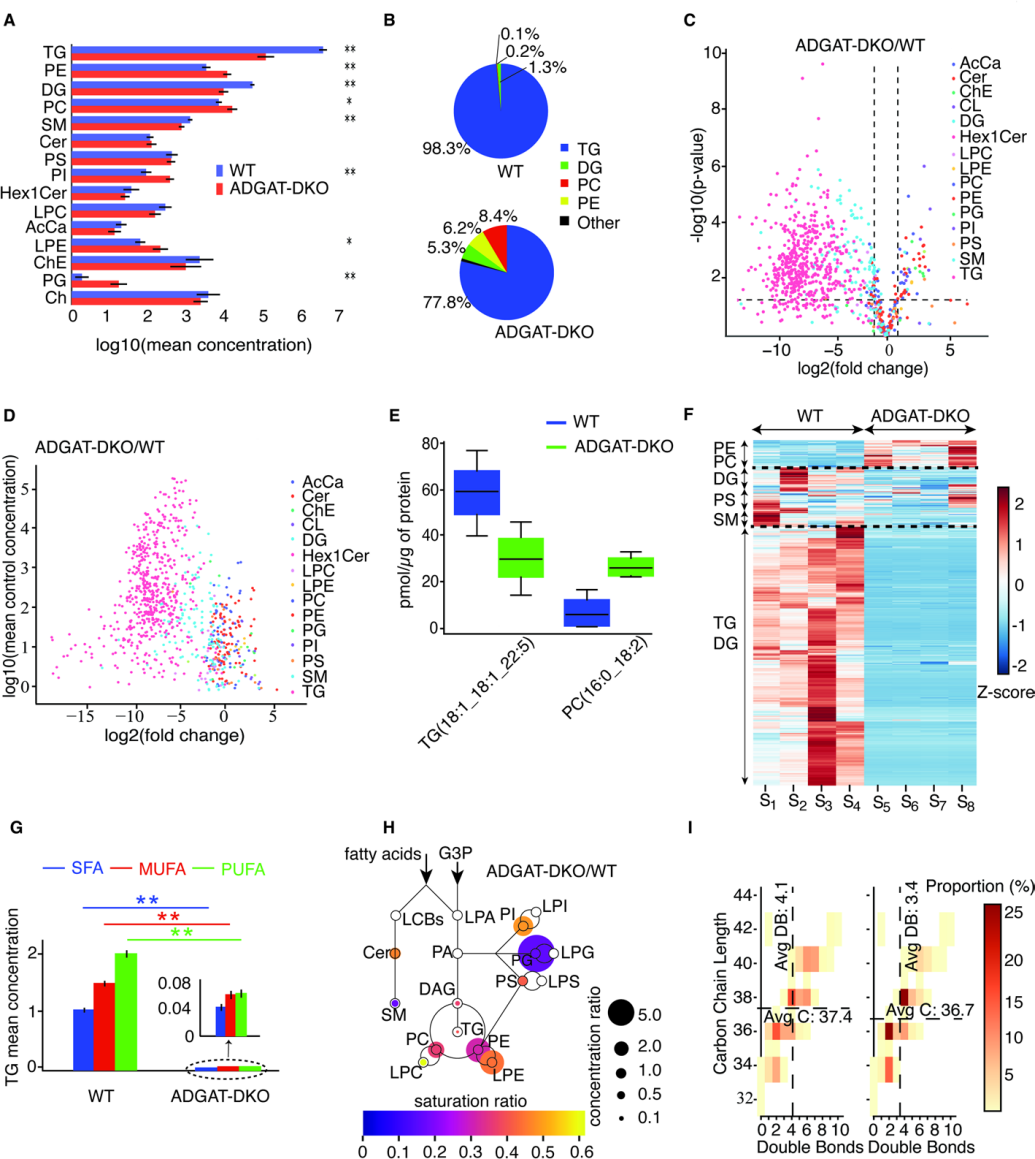
Differential lipid analysis of ADGAT-DKO mice. **A** Bar graphs comparing mean lipid class concentrations between WT and ADGAT-DKO mice. Statistical significance: *p < 0.05, **p < 0.01 and ***p < 0.001. **B** Pie charts showing the proportional distribution of lipid classes. **C** Volcano plot displaying log2 fold change versus statistical significance for individual lipid species. **D** Scatter plot of mean WT concentration versus fold change. **E** Box plot comparing concentration distributions of representative TG and PC species. **F** Lipidomic heatmap displaying z-score normalized concentrations for all lipid species, with hierarchical clustering. **G** Saturation profile analysis for TG species showing SFA, MUFA and PUFA composition. Statistical significance: *p < 0.05, **p < 0.01 and ***p < 0.001. **H** Metabolomic pathway visualization showing fold change (circle size) and saturation ratio (circle color). Warmer colors indicate higher saturation; white circles indicate undetected classes. **I** Fatty acid composition heatmap for the PE class, displaying lipid distribution by carbon chain length (y-axis) and double bond count (x-axis), with average markers overlaid for each condition

Figure 3 represents visualization outputs from *LipidCruncher*. Bar and pie charts (Fig. 3A and B**)** reveal that TG levels in ADGAT-DKO adipose tissue are reduced by ~ 96%, though TG remains the predominant class (decreasing from 98.3 to 77.8% of the total lipid composition). TG depletion is accompanied by increased phospholipid amounts (PC, PE, LPE) and decreased DG and SM levels.

The volcano plot (Fig. 3C) provides species–level comparison, showing multiple TG species significantly reduced while phospholipid species are elevated. A complementary scatter plot (Fig. 3D) illustrates the relationship between mean WT concentration and fold-change across lipid classes, revealing reduced TG and DG levels across both high- and low-abundance species. Box plots (Fig. 3E**)** enable examination of concentration distributions for individual lipid species across conditions.

The lipidomic heatmap (Fig. 3F**)** provides a high-resolution view of alterations across 942 lipid species, organized into three clusters: PE and PC lipids, DG, PS and SM species, and TG and DG lipids. Saturation profile analysis of TG species (Fig. 3G**)** reveals a shift from PUFAs to MUFAs in the ADGAT-DKO samples, suggesting altered fatty acid metabolism. The pathway visualization (Fig. 3H**)** captures dynamic changes in lipid class concentrations, illustrating the interplay between lipid biosynthesis and degradation pathways in the absence of DGAT enzymes.

The fatty acid composition heatmap for PE (Fig. 3I) reveals a reduction in average double-bond content (4.1–3.4) and carbon chain length (37.4–36.7) in ADGAT-DKO samples, indicating shifts toward more saturated, shorter fatty chains. These compositional changes reflect altered fatty acid availability driven by impaired TG synthesis.

## Implementation

*LipidCruncher* was developed in Python 3.11 and follows a layered architecture with strict dependency rules. Immutable Pydantic data models define the experimental configuration, normalization parameters, and statistical test settings. A services layer of stateless classes implements all business logic—including format detection, data cleaning, normalization, quality assessment, statistical testing, and visualization—with no dependency on the user interface framework. A workflow layer orchestrates multi-step pipelines by calling services in sequence. A single adapter class bridges the business logic to Streamlit’s session state and caching mechanisms, and thin UI modules handle rendering. This separation enables all analytical logic to be developed and tested independently of the web framework.

Accommodating heterogeneous input formats with divergent column structures, quality metrics, and naming conventions was addressed through a format-detection service that identifies the input type and delegates to format-specific cleaners, standardizing all inputs into a uniform internal DataFrame structure with consistent column naming. Lipid nomenclature is standardized to LIPID MAPS shorthand notation [34] through dedicated parsing and formatting utilities that reconcile notation differences across platforms. We addressed application performance by implementing Streamlit’s built-in caching mechanisms to store the results of expensive computations (data cleaning, normalization, statistical tests), avoiding redundant recalculation when users adjust visualization parameters or navigate between modules. Containerizing the application with Docker resolved environment-dependent deployment behavior, and the application is deployed on Amazon Web Services (AWS) using Elastic Container Service (ECS).

The web interface was built with Streamlit to provide a guided, no-code workflow accessible to researchers without programming experience. Interactive visualizations were implemented using Plotly (supporting hover, zoom, and filtering) with Matplotlib/Seaborn for static publication-quality heatmaps. Statistical analyses use scipy.stats for hypothesis testing (t-tests, Mann-Whitney U, ANOVA, Kruskal-Wallis), statsmodels for multiple testing corrections (Benjamini-Hochberg, Bonferroni) and post-hoc tests, and scikit-learn for PCA with data preprocessing. Data manipulation relies on pandas and numpy, and PDF report generation uses reportlab. The codebase includes a comprehensive test suite covering unit, integration, and UI tests. The source code is available under an open-source license on GitHub. Detailed analytical and methodological specifications are provided in Supplementary Methods.

## Conclusions

Here we present *LipidCruncher*, an open-source platform that simplifies analysis of lipidomic data by integrating quality assessment, visualization, and statistical tools into a seamless workflow. Designed to accommodate diverse mass spectrometry outputs, it enables researchers to ensure sufficient quality of the data, bypass technical bottlenecks, and focus on extracting biological meaning from complex datasets. The built-in quality control safeguards ensure data integrity, minimizing errors and enhancing reproducibility.

*LipidCruncher* and existing tools serve complementary functions. *MetaboAnalyst* [13] excels in raw spectra processing and advanced multivariate analytics. Among specialized platforms, *lipidr* [10] provides lipid set enrichment analysis, *LipidSig* [11] offers machine learning and network analysis, *ADViSELipidomics* [12] enables batch correction for multi-site studies, and *LipidOne* [14] focuses on building-block level analysis with biomarker discovery. *LipidCruncher* addresses a distinct need: providing a streamlined, guided workflow that tightly integrates quality control with normalization. Users can immediately assess the effect of normalization through box plots and BQC analysis, iteratively remove high-CoV species with selective restore, and identify outlier samples through PCA-guided removal, all within a single continuous workflow. The platform further provides comprehensive lipid-specific visualization and straightforward statistical testing with a two-level multiple testing correction framework. While tools such as *MetaboAnalyst* offer a broader range of normalization methods and advanced multivariate analytics, *LipidCruncher* focuses on the approaches most commonly used in lipidomics workflows and prioritizes accessibility for researchers without bioinformatics expertise. For specialized analyses beyond *LipidCruncher*’s scope—such as pathway enrichment, machine learning, or multi-omics integration—researchers can leverage the dedicated platforms designed for these purposes. This division of capabilities allows each tool to excel at its intended function while collectively serving the broader needs of the lipidomics research community.

Current limitations of *LipidCruncher* merit consideration. While the platform provides multiple quality checkpoints, it cannot identify all potential problems, particularly in upstream processes (peak integration and lipid identification). Regarding lipid identification errors, *LipidCruncher*’s retention time analysis may detect gross misclassifications (e.g., a triacylglycerol incorrectly identified as a phospholipid), but structural misidentifications within the same lipid class (e.g., incorrect fatty acid composition assignments) cannot easily be detected. *LipidCruncher* standardizes lipid nomenclature but does not validate whether reported lipid species are biologically plausible; this responsibility remains with the user. Additionally, while the platform supports outputs from major lipidomics software (*LipidSearch* [6], *MS-DIAL* [9], *Metabolomics Workbench* [17]), it does not directly accommodate all processing tools (e.g., *mzMine* [15] or *Skyline* [31]). Users working with these platforms must standardize their data into the generic CSV format, requiring manual formatting before analysis. Future development will expand format support and incorporate additional quality validation steps.

## Availability and requirements

Project name: LipidCruncher; Project home page: https://lipidcruncher.org/; Source code: https://github.com/FareseWaltherLab/LipidCruncher; Operating system(s): Platform independent (web-based); Programming language: Python 3.11; Other requirements: Modern web browser (Chrome, Firefox, Safari, or Edge recommended); License: MIT License; Any restrictions to use by non-academics: None.

Lipid names follow the LIPID MAPS shorthand notation [34]: Class chain1_chain2, where the class abbreviation is followed by a space (not parentheses), and each chain is denoted as X: Y, with X representing the number of carbon atoms and Y representing the number of double bonds (e.g., PC 16:0_18:1). For sphingolipids, the long-chain base and N-acyl chain are separated by a forward slash (e.g., Cer 18:1;O2/24:0). Hydroxyl groups are indicated using the element-before-count convention (e.g., ;O2 for two hydroxyl groups). Lysophospholipid species are represented with a single chain (e.g., LPC 20:0). For glycerolipids and phospholipids with multiple chains, chains are ordered by ascending carbon count, then ascending double bond count. Consolidated format represents total chain composition (e.g., PC 34:1).

## Supplementary Information

Below is the link to the electronic supplementary material.

Supplementary Material 1

Supplementary Material 2

Supplementary Material 3

Supplementary Material 4

Supplementary Material 5

Supplementary Material 6

## Data Availability

The ADGAT-DKO lipidomic datasets analyzed in the case study demonstration are provided as supplementary materials: Supplementary File 1 contains the normalized data in generic format, and Supplementary File 2 contains the raw *LipidSearch* output. Supplementary Files 3 and 4 contain publicly available third-party datasets included to demonstrate format capability. Supplementary Files 3 is adapted from the MS-DIAL lipidome atlas [32], available under the original publication’s data sharing terms. Supplementary File 4 is from Metabolomics Workbench (Study ID: ST001323) [33], available under Creative Commons Attribution License (CC BY 4.0).All these datasets are also available on the [Farese and Walther lab GitHub](https:/github.com/FareseWaltherLab/LipidCruncher) site.

## Abbreviations

BQC: Batch quality control
CoV: Coefficient of variation
DGAT1: Diacylglycerol acyltransferase 1
DGAT2: Diacylglycerol acyltransferase 2
LC-MS/MS: Liquid chromatography coupled to mass spectrometry
PCA: Principal component analysis
WT: Wild type
ADGAT-DKO: Adipose-specific DGAT double knockout
SFA: Saturated fatty acids
MUFA: Mono-unsaturated fatty acids
PUFA: Poly-unsaturated fatty acids
PC: Phosphatidylcholine
LPC: Lysophosphatidylcholine
PE: Phosphatidylethanolamine
LPE: Lysophosphatidylethanolamine
PI: Phosphatidylinositol
LPI: Lysophosphatidylinositol
PS: Phosphatidylserine
LPS: Lysophosphatidylserine
PG: Phosphatidylglycerol
LPG: Lysophosphatidylglycerol
PA: Phosphatidic acid
LPA: Lysophosphatidic acid
CL: Cardiolipin
CDP-DAG: Cytidine diphosphate diacylglycerol
TG: Triacylglycerol
DG: Diacylglycerol
MAG: Monoacylglycerol
Cer: Ceramide
SM: Sphingomyelin
HexCer: Hexosylceramide
CerG1: Monoglycosylceramide
CerG2: Diglycosylceramide
CerG3: Triglycosylceramide
LCB: Long-chain base
ChE: Cholesteryl ester
CE: Cholesteryl ester
Ch: Cholesterol
AcCa: Acyl carnitine
